# Arthroscopic Rotator Cuff Repair In Patients With Parkinson’s Disease: A Propensity Score Matching Study With Minimum 2-Year Follow-up

**DOI:** 10.1186/s12891-022-06007-z

**Published:** 2022-12-05

**Authors:** Yufan Qian, Kailun Wu, Feng Zhou, Li Li, Jiong Jiong Guo

**Affiliations:** 1 Department of Orthopedics and Sports Medicine, The First Affiliated Hospital of Soochow University, 188 Shizi Street, Suzhou, 215006 People’s Republic of China; 2 Department of Orthopedics, Dushu Lake Hospital Affiliated to Soochow University, Suzhou, People’s Republic of China; 3 Department of Rehabilitation, The First Affiliated Hospital of Soochow University, 188 Shizi Street, Suzhou, 215006 People’s Republic of China

**Keywords:** Rotator cuff repair, Arthroscopic repair, Parkinson’s disease, Clinical outcome

## Abstract

**Background:**

Although the effectiveness of arthroscopic rotator cuff repair (ARCR) for structural and functional outcomes has been widespread proven, few researchers investigated the impact of ARCR on patients with Parkinson’s Disease (PD), which may have previously been viewed as a relative contraindication to ARCR.

**Methods:**

Data were collected retrospectively for all patients who underwent ARCR for small- to large-sized rotator cuff tears between September 2014 and May 2019. Patients were eligible for the study if they indicated that they diagnosed with rotator cuff repair and had minimum 2-year postoperative outcome scores for the range of motion (ROM), the Western Ontario Rotator Cuff Index (WORC), the Constant-Murley Score (CMS), the University of California, Los Angeles (UCLA), Pittsburgh Sleep Quality Index (PSQI), Hospital Anxiety and Depression Scale (HADS), and the visual analog scale (VAS) for pain. Propensity score matching (PSM), a statistical method, was used to screen a control group without PD matched 1:1 with similar age, sex, tear size, preoperative stiffness, and fatty infiltration, which have previously been identified as important factors influencing success rates.

**Results:**

Three hundred and eighty-nine patients met all study criteria including required follow-up, of whom 31 and 358 with PD and without PD, respectively. After adjusting for confounders, the propensity score matched indicators were compared, patients with PD experienced significantly more pain (4.45 ± 2.43 vs. 0.52 ± 1.18; *P*<.001) and had lower WORC (49.10 ± 21.22 vs. 78.90 ± 17.54; *P*<.001), CMS (46.77 ± 22.24 vs. 79.45 ± 14.74; *P*<.001) and UCLA (21.11 ± 8.54 vs. 28.16 ± 6.16; *P*<.001) scores respectively than the matched control group. They also exhibited higher sleep disturbance (10.04 ± 5.36 vs. 5.19 ± 3.28; *P*<.001), as well as higher anxiety and depression psychological status at 24 months (*P*<.001; *P*<.001). Overall clinical outcomes from preoperatively to postoperatively were not improved significantly for patients with PD vs. without PD.

**Conclusion:**

Patients with PD experienced significantly more pain, resulted in worse shoulder functional outcomes, and reported persistently diminished mental and physical health status. Shoulder surgeons should be cognizant of PD as an outcome-modifying variable when treating patients with rotator cuff tears. This finding suggested that the need for ARCR in patients with PD should be carefully considered in the light of personalized needs and physical conditions.

## Introduction

Parkinson’s disease (PD) is a progressive age-related progressive neurological disease which has long been characterized by the appearance of a plethora of motor features such as bradykinesia, muscular rigidity, resting tremor, and postural and gait impairment [1]. Additionally, the non-motor sequelae such as cognitive deterioration, depressive symptoms and impairment of the autonomic nervous system, may reduce quality of life even before the motor symptoms [2]. These classical symptoms are closely associated with Lewy bodies pathology and early prominent death of dopaminergic neurons in the substantia nigra pars compacta (SNpc) [3]. According to the epidemiological survey, PD was the most prevalent neurodegenerative disorder after Alzheimer’s disease, which aggravates social welfare and economic burden with the emergence of an aging society [4]. In industrialized countries, the prevalence of the disease was generally estimated to be 0.3% of the entire population, rising to approximately 1-2% over the age of 65 [4, 5].

Besides the effects of the disease itself, musculoskeletal comorbidities were an equally key factor in the deterioration of quality of life and physical functioning in PD. Frozen shoulder was originally thought to be a possible presenting feature of PD, and patients in this subgroup had 21 times the odds ratio of sufferring from shoulder discomfort compared to the controls [6, 7]. There is a consensus that tendon tearing, especially supraspinatus tendon, is the most typical feature of shoulder abnormalities in this group [8, 9]. Many patients with PD had thoracic kyphosis and reduced trunk mobility, they could develop shoulder impingement syndrome and arthritis of the capsule [10]. Patients with mild and severe PD had significantly lower acromion distance (a measure of the width of the subacromial space) compared to healthy controls [9]. Postoperative outcomes of common orthopedic problems in these patients have been partially controversial because of conflicting reports on functional improvement in the literature [11–15]..

Quality of life in Parkinson’s patients is affected by rotator cuff tears. However, as far as we knew, there were no relevant descriptions of patients with Parkinson's disease undergoing ARCR in the past literature. As far as we knew, this study was the first to investigate whether these musculoskeletal comorbidities in Parkinson’s patients affect the success of ARCR. We hypothesized that Parkinson’s patients would have worse outcomes compared to patients without PD.

## Materials and methods

### Patients selection

This retrospective study was conducted at *blind the name* with the approval of the Institutional Review Board Hospital. In all cases rotator cuff tears were diagnosed previously by clinical examination, preoperative relevant imaging, and reconfirmed arthroscopically. Patients were recommended for surgery if their persistent shoulder discomforts were unresponsive to conservation treatment protocol (ie, medication, injection or physiotherapy) for a minimum observation period of 8 weeks. For inclusion in the study all patients had been formally evaluated by a neurologist and have a minimum follow-up period of 2 years. The Hoehn and Yahr (H&Y) scale was utilized to quantitatively assess the severity of their preoperative symptoms [16]. To ensure that the comparisons in this retrospective study were made under the most homogeneous conditions possible, patients with mild to moderate PD (H&Y stage I-II) and small- to large-sized tears were screened to avoid the confounding influence of non-healing resulting from poor tendon quality, which occurs frequently in massive-sized tears. In addition, other exclusion criteria were patients with partial-thickness tear, irreparable massive or acute trauma-related tear of the supraspinatus tendon, revision rotator cuff procedures, degenerative arthritis of ipsilateral glenohumeral joint, Workers’ Compensation claims, or a history of previous surgery on the ipsilateral shoulder.

### Surgical technique

All procedures were performed with the patient under the condition of general anesthesia in the beach-chair position by a single senior shoulder surgeon. After assessing the stiffness of the shoulder, manual manipulation for release was performed before arthroscopic repair. The operated limb was immobilized in traction by using of a sleeve with 3 kg weights. The entire diagnostic procedures involved exploring the glenohumeral joint and clearing the hyperplastic synovium. Biceps tenotomy was carried out in the case of concomitant long head biceps pathology or instability. If there were signs of severe stenosis in the subacromial space, an arthroscopic subacromial decompression was performed to create a flat subacromial surface. The footprint was grinded to expose cancellous bone and ooze blood. Grasping the retracted tendon to assess elasticity. If any sign of subscapularis tendon tear was found intraoperatively, the first step was to perform a single or double row suture of the subscapularis tendon, depending on the size of the tear. Thereafter, two medial-row suture anchors were first inserted medial to the footprint. Using tissue penetration tools, the ends of each suture were passed through the tendon, retaining as much residual length as possible. Two lateral anchors were inserted laterally at the margin of the footprint, obtaining the maximum area of tendon-to-bone interface apposition. The repair was finished by knotting the simple suture in the lateral row and tying the suture in the medial row in a mattress fashion. Finally, the sutures were cut flush.

### Postoperative rehabilitation

#### First phase (immediate postoperative period to 4 weeks)

From the day of operation, all patients who underwent ARCR were instructed to follow a standard postoperative rehabilitation program, with shoulders immobilized in an abduction brace. During fixation, the patients conducted the exercise of muscle contraction and performed gentle passive motions, comprising pendulum exercises, assisted flexion and extension exercises. Typically, we encouraged patients to remove the shoulder bracket several times a day, once for daily activities.

### Second phase (postoperative weeks 5 to 12)

With gradual removal from the brace at this stage, the patient was instructed to perform isometric contractions at different angles below the plane of the shoulder joint and closed chain training.

#### Third phase (postoperative months 3 to 6)

At this time, rotator cuff strengthening exercises were introduced gradually. The reasonable timing of the onset of strengthening was mainly based on the healing of the tendon. The patients were permitted to practice light activities. Full return to labor requiring high muscular endurance and shoulder stability may take up to 6 months.

#### Clinical outcomes and radiological characteristics

Baseline characteristics including patient demographics (age, gender, duration, involvement of dominant arm) and other underlying diseases were recorded in existing medical documents. All patients underwent a thorough shoulder examination as well as radiological diagnosis, including radiographs (anteroposterior view, supraspinatus outlet view) and magnetic resonance imaging (MRI) of the involved shoulder preoperatively. The tear pattern of rotator cuff, as assessed on MRI preoperatively, was recorded under direct arthroscopic visualization. The anterior-posterior (AP) length of the tear was classified as small (<1 cm), medium (1-3 cm), large (3-5 cm), or massive (>5 cm) on the basis of the rating system proposed by DeOrio and Cofield [17]. After debriding the edge of tear end, the maximal AP length of the tear was measured arthroscopically using a graduated probe. Since the medial-lateral (ML) size of the tear varies considerably due to subtle position differences, we assessed ML length on preoperative MRI with a T2-weighted image. According to the classification proposed by Patte [18], tendon retraction was assessed on coronal T2 fat-saturated images (not retracted, grade 1; retracted to humeral head, grade 2; or grade 3, retracted to glenoid). Fatty degeneration of the supraspinatus, subscapularis, infraspinatus, and teres minor tendons was determined according to the “Y view” of T2-weighted images by MRI [19] (no fat infiltration classified as grade 0, some fatty streaks classified as grade 1, more muscle than fat classified as grade 2, equal amounts classified as grade 3, more fat than muscle classified as grade 4). The global fatty degeneration index (GFDI) was then calculated, representing the average level of overall muscle fat degeneration.

Comprehensive shoulder physical results were conducted and collected by an independent blinded assessor pre- and postoperatively. The average pain assessment is measured using a visual analogue scale (VAS), which is a 10 cm horizontal line. The left side represents “no pain at all”, while the right side represents “the most severe pain”. The Western Ontario Rotator Cuff (WORC) Index [20], Constant-Murley Score (CMS) [21] and the University of California, Los Angeles (UCLA) [22] scale are used for the comprehensive assessment of shoulder mobility as well as pain. As for the WORC score, we used the percentage system, with larger scores indicating better function. The Hospital Anxiety and Depression Scale (HADS) [23] consists of two 7-item subscales measuring anxiety (HADS-A) and depression (HADS-D). It is commonly used as a screening modality to assess anxiety and depression in people with musculoskeletal disorders. Each item on the questionnaire is scored from 0 to 3. The final scores for anxiety and depression therefore range from 0 to 21, with higher scores indicating a greater likelihood of anxiety or depression. The Pittsburgh Sleep Quality Index (PSQI) [24] is a 19-question questionnaire for self-assessment to measure the quality of sleep of patients. Higher scores indicate poorer sleep quality. Shoulder ROM of forward flexion and abduction were measured with a goniometer. For internal rotation measurements, patients were instructed to use their thumb to reach as high as possible on the spine. In order to be statistically viable for internal rotation, according to Cho et al [25], we converted values into contiguously numbered groups: 1 through 12 represented T1 through T12; 13 through 17 represented L1 through L5; 18 represented sacrum; and 19 represented buttock. Shoulder stiffness was defined as forward elevation <120°, external rotation with the arm at the side at <30°, or internal rotation at a level lower than L3 [26].

### Statistical analysis

For the statistical analysis, descriptive statistics were calculated and reported as means, standard deviations, ranges, and percentages. Student *t* test, Fisher exact test and the *x*^2^ test were implemented for continuous and categorical variables, respectively, to compare baseline characteristics of demographic, clinical and radiological factors before and after matching. Independent-samples *t* test and paired-samples *t* tests were used to compare differences in motor and functional scores within and between groups pre- and post-operatively. PSM, a statistical method, was used to screen a control group without PD matched 1:1 with similar age, sex, tear size, preoperative stiffness, and fatty infiltration, which have previously been identified as important factors influencing success rates. In addition, binary regression logic analysis was used to determine the influencing factors of postoperative satisfaction. The IBM SPSS version 26.0 (IBM SPSS Statistics for Windows, version 26.0) was used for all the statistical analyses. The confidence level was assumed to be 95%, with the significance level set at *p=*0.05.

## Results

### Demographic and clinical characteristics

**Fig. 1:**
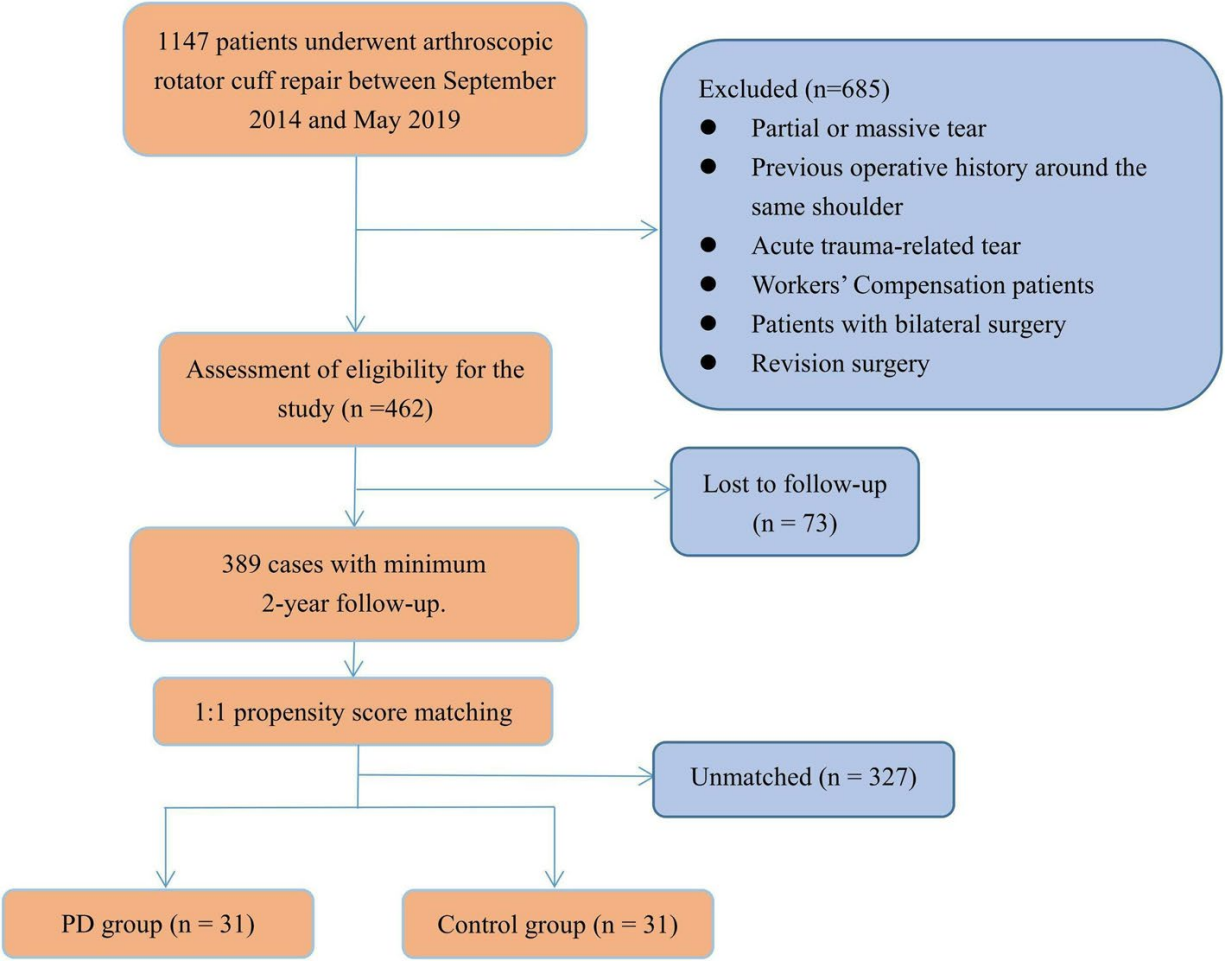
Patient selection flowchart

During the research period, 389 patients qualified for the inclusion criteria, of which 316(81%)had followed up for at least 2 years. The patient selection flowchart is depicted in Figure 1. Prior to propensity score matching, the mean ages were 63.8±6.0 and 601.0±7.6 years, respectively. There were 13 male and 18 female with PD and 153 and 205 without PD. For the other matching variables, there was no statistically significant difference between group comparisons, except for preoperative stiffness (*P* = .045) (Table 1). The baseline characteristics of the PD group versus the control group are shown in Table 1.

**Table 1:** Demographic and baseline clinical characteristics. Before and After Propensity Score Matching^*a*^

	Before Matching			After Matching		
	PD (*n =* 31)	Control (*n* = 358)	*P* Value	PD (*n =* 31)	Control (*n =* 31)	*P* Value
Age at surgery, y	63.8 ± 6.0	61.0 ± 7.6	**.041**	63.8 ± 6.0	61.1 ± 7.4	.117
Male:female	13:18	153:205	.931	13:18	14:17	.798
Dominant hand, n (%)	19(61.3)	191(49.6)	.395	19(61.3)	15(48.4)	.307
Duration of symptoms, mo	12.7 ± 4.6	12.3 ± 5.5	.686	12.7 ± 4.6	12.7 ± 5.1	.979
Preoperative stiffness	10(32.3)	63(17.5)	**.045**	10(32.3)	10(32.3)	1.000
Smoking, n (%)	8(25.8)	107(29.8)	.633	8(25.8)	11(35.5)	.409
Diabetes mellitus, n (%)	4(12.9)	71(19.8)	.348	4(12.9)	5(16.1)	1.000
Dyslipidemia, n (%)	6(19.4)	96(26.8)	.365	6(19.4)	7(22.6)	.755
Tear size^*b*^						
AP dimension, mm	24.5 ± 10.7	24.6 ± 8.4	.964	24.5 ± 10.7	22.8 ± 9.8	.508
Retraction, mm	21.9 ± 7.7	21.6 ± 7.8	.842	21.9 ± 7.8	23.2 ± 9.2	.563
GFDI^*c*^	1.69 ± 0.59	1.57 ± 0.70	.362	1.69 ± 0.59	1.70 ± 0.85	.954
Follow-up period, mo	30.2 ± 4.2	32.7 ± 6.4	**.004**	30.2 ± 4.2	34.1 ± 7.0	**.010**

^*a*^Data are provided as mean ± SD or n (%), unless otherwise noted. Bold indicates *P* < .05. *PD*, Parkinson’s Disease; *AP*, anteroposterior; *GFDI*, global fatty degeneration index

^*b*^Tear size was measured arthroscopically with a calibrated probe at the time of surgery and classified according to the rating system of DeOrio and Cofield

^*c*^Fatty infiltration was graded in accordance with the criteria established by Goutallier et al.

With a 1:1 ratio of PSM, statistical comparisons were able to be performed at similar baseline levels for all variables, including the preoperative stiffness (*P* = 1.000) (Table 2). Ultimately, 31 patients in each group who met the criteria were enrolled for further precise analysis. The measured AP length of tear was 24.5 ± 10.7 mm and retraction was 21.6±7.8 mm. Overall follow-up periods were 30.2±4.2 months for the PD group and 32.7 ±6.4 months for the control group (*P* = .004).

**Table 2:** Preoperative and postoperative active range of motion. Before and After Propensity Score Matching^*a*^

Outcome Measure	Before PSM			After PSM		
	PD Group	Control Group	*P* value	PD Group	Control Group	*P* value
ROM						
Forward-flexion, deg						
Preoperative	82.10 ± 20.53	78.95 ± 26.18	.429	82.10 ± 20.53	75.32 ± 26.52	.265
Postoperative	88.39 ± 36.18	146.01 ± 27.72	**< .001**	88.39 ± 36.18	140.65 ± 28.98	**< .001**
*P* value	.383	**< .001**		.383	**< .001**	
Abduction, deg						
Preoperative	73.87 ± 19.65	67.89 ± 23.36	.167	73.87 ± 19.65	66.94 ± 22.76	.204
Postoperative	84.52 ± 35.01	128.98 ± 27.72	**< .001**	84.52 ± 35.01	120.97 ± 30.83	**< .001**
*P* value	.112	**< .001**		.112	**< .001**	
Internal rotation^*b*^						
Preoperative	14.29 ± 2.40	15.57 ± 1.74	**< .001**	14.29 ± 2.40	16.10 ± 1.89	**.002**
Postoperative	13.94 ± 4.59	9.38 ± 4.07	**< .001**	13.94 ± 4.59	10.84 ± 4.24	**.008**
*P* value	.672	**< .001**		.672	**< .001**	

^*a*^Data are provided as mean ± SD, unless otherwise noted. Bold indicates *P* < .05. *PSM* Propensity score matching, *PD* Parkinson’s Disease, *ROM* Range of motion

^*b*^For ease of statistical analysis of internal rotation, the vertebral level was converted to a number as follows: T1 through T12 to 1 through 12; L1 through L5 to 13 through 17; sacrum to 18; and buttock to 19

### Functional assessment and mobility outcomes

**Fig. 2:**
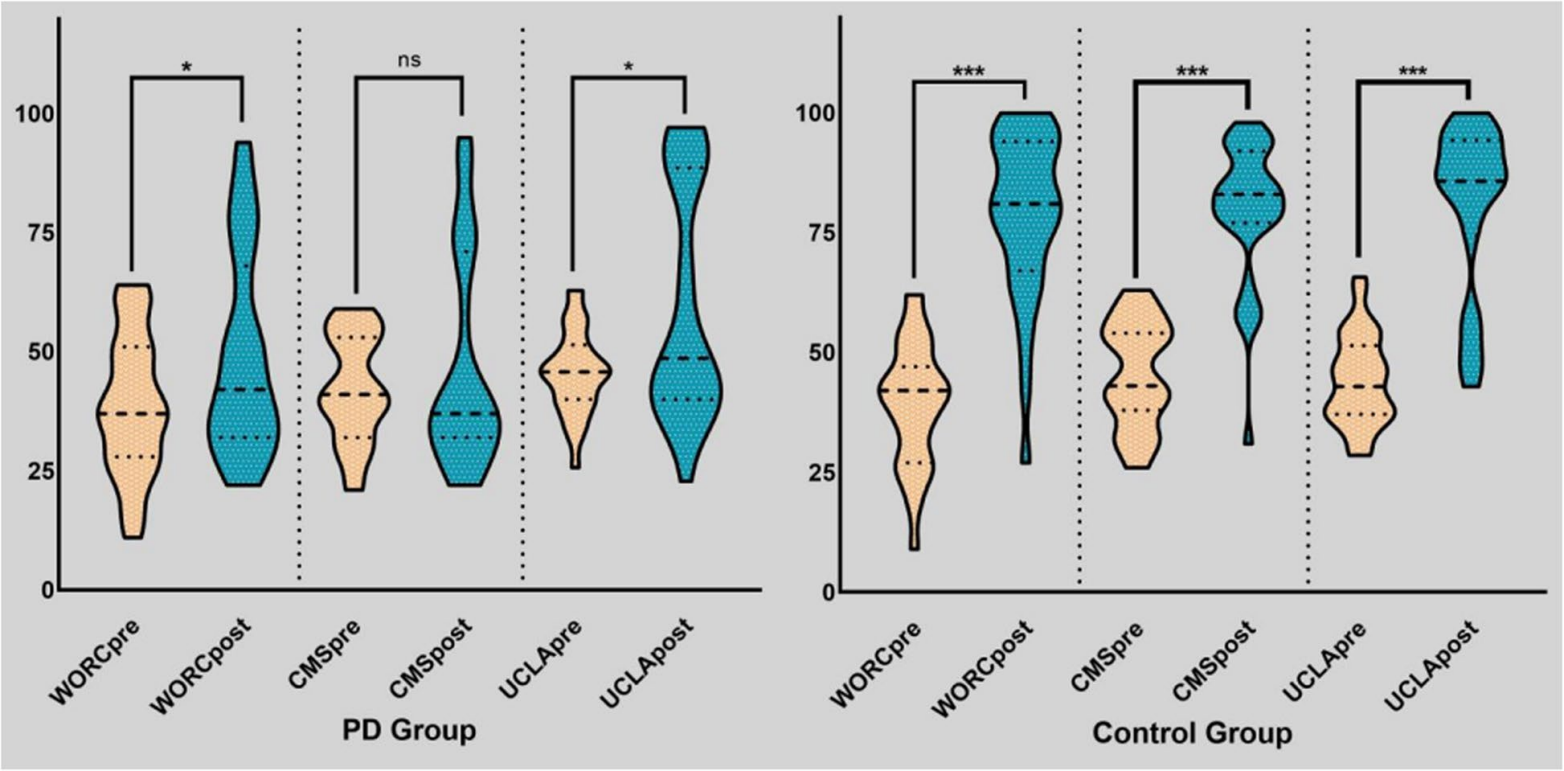
Shoulder functional scores. For consistency of data, we converted the score of shoulder joint function into a percentage scale. * *P* < .05; ***P < .001

**Fig. 3:**
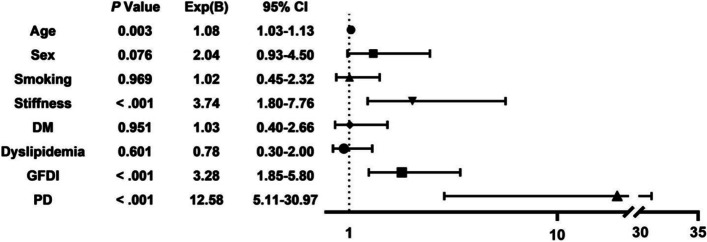
The frost plot of the postoperative satisfaction after ARCR

The groups did not differ significantly in preoperative forward flexion, abduction, VAS, WORC, CMS, UCLA, PQSI, HADS-A, or HADS-D. However, the PD group had significantly better preoperative internal rotation (*P*=.002). In the PD group, significant improvements from preoperatively to final follow-up were seen in WORC, UCLA and PQSI. The pain VAS, ROM, CMS and PQSI assessments at least 2 years postoperatively did not showed any differences between the matched groups (Table 3). Satisfaction with the surgical results among PD patients was poor for 11 (35.5%), fair for 7 (22.6%), good for 6 (19.4%), and very good for 7 (22.6%). In the matched control group, significant improvements were found in ROM, VAS, various functional outcomes and PQSI. In an intergroup comparison pre- to postoperatively, the matched control group had significantly greater improvement in forward flexion, abduction, internal rotation, VAS, WORC, CMS, UCLA, and PQSI compared with patients with PD (Figure 2, Table 2, Table 3). We classified “poor” and “fair” as unsatisfactory outcomes, and “good” and “very good” as satisfactory outcomes. A binary regression analysis of postoperative satisfaction was performed in 389 patients. Age (*P*=.003), preoperative stiffness (< .001), fatty infiltration (< .001) and Parkinson's disease (< .001) were found to be independent risk factors affecting patient satisfaction (Figure 3).

**Table 3:** preoperative and postoperative functional outcome scores. Before and After Propensity Score Matching^*a*^

Outcome Measure	Before PSM			After PSM		
	PD Group	Control Group	*P* value	PD Group	Control Group	*P* value
VAS						
Preoperative	5.35 ± 1.05	5.13 ± 1.28	.345	5.35 ± 1.05	4.77 ± 1.41	.071
Postoperative	4.45 ± 2.43	0.68 ± 1.17	**< .001**	4.45 ± 2.43	0.52 ± 1.18	**< .001**
*P* value	.054	**< .001**		.054	**< .001**	
WORC						
Preoperative	38.55 ± 14.62	40.62 ± 14.90	.458	38.55 ± 14.62	39.32 ± 12.75	.825
Postoperative	49.10 ± 21.22	83.10 ± 15.26	**< .001**	49.10 ± 21.22	78.90 ± 17.54	**< .001**
*P* value	**.004**	**< .001**		**.004**	**< .001**	
CMS						
Preoperative	42.13 ± 11.10	47.66 ± 12.02	**.014**	42.13 ± 11.10	45.45 ± 10.93	.240
Postoperative	46.77 ± 22.24	82.20 ± 12.99	**< .001**	46.77 ± 22.24	79.45 ± 14.74	**< .001**
*P* value	.262	**< .001**		.262	**< .001**	
UCLA						
Preoperative	15.67 ± 3.05	16.20 ± 3.92	.714	15.67 ± 3.05	15.45 ± 3.33	.554
Postoperative	21.11 ± 8.54	29.44 ± 5.49	**< .001**	21.11 ± 8.54	28.16 ± 6.16	**< .001**
*P* value	**.002**	**< .001**		**.002**	**< .001**	
HADS-D						
Preoperative	5.65 ± 2.17	6.02 ± 2.62	.443	5.65 ± 2.17	5.65 ± 2.12	1.000
Postoperative	5.16 ± 3.42	2.14 ± 1.93	**< .001**	5.16 ± 3.42	1.52 ± 1.65	**< .001**
*P* value	.275	**< .001**		.275	**< .001**	
HADS-A						
Preoperative	6.31 ± 2.52	6.43 ± 2.82	.833	6.31 ± 2.52	6.68 ± 2.54	.583
Postoperative	5.58 ± 3.97	2.47 ± 2.38	**< .001**	5.58 ± 3.97	2.52 ± 2.53	**.001**
*P* value	.401	**< .001**		.401	**< .001**	
PQSI						
Preoperative	12.52 ± 3.29	12.89 ± 3.45	.560	12.52 ± 3.29	11.32 ± 4.24	.220
Postoperative	10.13 ± 5.30	5.85 ± 3.47	**< .001**	10.13 ± 5.30	5.19 ± 3.28	**< .001**
*P* value	**.025**	**< .001**		**.025**	**< .001**	

^*a*^Data are provided as mean ± SD, unless otherwise noted. Bold indicates *P* < .05. *PSM*, propensity score matching; *PD* Parkinson’s Disease, *VAS* Visual analog scale, *WORC* Western Ontario Rotator Cuff Index, *CMS* Constant-Murley Score, *UCLA* University of California, Los Angeles, *HADS-A* Hospital Anxiety and Depression Scale anxiety subscale, *HADS-D* Hospital Anxiety and Depression Scale depression subscale, *PQSI* Pittsburgh Sleep Quality Index

According to the Hoehn and Yahr classification [16], the latest follow-up disability was classified as stage 1 in 24 patients (77.42%), stage 2 in 7 patients (29.17%). The latest follow-up disability was classified as stage 1 in 17 patients (54.84%), stage 2 in 11 patients (35.48%), stage 3 in 2 patients (6.45%), stage 4 in 1 patient (3.23%). In 21 patients (67.74%), the disability classified at the same level the previous level. 9 and 1 patients had deteriorated in disability by 1 and 2 stage 29.03% and 3.23%) (Table 4).

**Table 4:** Patient’s preoperative and latest follow-up stage of parkinson’s disease, According to Hoehn and Yahr Scale

		Number of patients	
Stage		Preoperative	Latest Follow-up
I	Unilateral involvement	24	17
II	Bilateral involvement without impairment of balance	7	11
III	Mild to moderate bilateral disease; some postural instability; physically independent	0	2
IV	Severe disability; still able to walk or stand unassisted	0	1
V	Confined to wheelchair	0	0

## Discussion

When compared with a propensity-matched control group, patients with PD had worse self-reported outcome scores and the improvement of shoulder mobility did not reach statistical significance. Shoulder pain in these patients became irregular, and this persistence was closely related to sleep disturbances and poor psychological status. Koh et al [8] reported that ultrasonography abnormalities of the shoulder joint were common in patients with early stage PD. The results revealed that the majority of the patients (70%) had rotator cuff tendon tearing, most frequently seen at the supraspinatus and subscapular tendons, with a small proportion (26.4%) having concomitant adhesive capsulitis with signs of shoulder stiffness. Yucel and Kusbeci [9] investigated shoulder pathologies in PD patients by using MRI. In addition to tendons and joint capsule changes, there is remodelling of bone structures (cortical irregularity, edema, cyst formation). Riley et al [6] found that periarthritis of shoulder occurred more often prior to, or simultaneously with, the onset of PD than after disease onset. Adhesive capsulitis of shoulder joint should be recognized as a presenting symptom of PD. This is consistent with what we found in our study, where the probability of preoperative rotator cuff tears combined with stiffness in PD patients differed from controls. Muscle rigidity is an important factor in the occurrence of adhesions, especially during postoperative rehabilitation. In addition, the slight friction effect of tremor may be a mechanical factor for poor healing of the tendon-bone interface.

This study identified significant individual differences in the functional outcomes of patients with PD after ARCR. There were significant differences with matched control group. However, there was a still paucity of high-level consensus to underpin surgeons in decision-making and perioperative consultations, particularly with reference to the prognosis of ARCR in this neglected subgroup. Joint procedures in patients with PD are controversial, and functional improvement needs to be viewed separately from pain relief. Koch et al [11] first analyzed the effectiveness of total shoulder arthroplasty in patients with mild to moderate PD. Patients had relief of pain postoperatively and overall functional results were surprisingly poor, particularly in patients over age 65. In addition, higher dissatisfaction [12] and significantly higher incidence of complications [26] were observed. Oni and Mackenney [13] initially even recommended that total knee arthroplasty should be contraindicated in PD patients due to all patients had complications of hamstring rigidity and flexion contractures. Recent studies have suggested that total knee or hip arthroplasty may achieve equivalent benefits in terms of efficacy, survivorship and complication rate in Parkinson's disease patients [15, 27]. Depressive and anxious states are common mood disorders in patients with PD, and exacerbations of these psychological states are associated with poorer quality of life [28]. We found that psychological depression and anxiety may result from less than expected recovery from postoperative shoulder symptoms. This led to a stagnation of postoperative shoulder activity, a vicious circle exacerbated. Our binary regression analysis of patient satisfaction showed that patients with Parkinson's disease were 12 times more likely to be dissatisfied than normal patients much higher than other independent risk factors such as age, preoperative stiffness, and fatty degeneration. As joint stiffness and pain continued to affect life, patients without psychological intervention showed more psychological disorders, poorer sleep quality, and social resistance.

Unlike normal patients, physical pain in patients with PD was more difficult to manage due to its diversity and complexity. In view of our findings above, we believed that surgical treatment could hardly achieve satisfactory results. We recommended that the PD should be managed preoperatively, including regular neurological evaluation and standard treatment. Ideally, disease progression in PD should be managed promptly and with minimal shoulder stiffness. Surgeons should pay close attention to the dynamic changes of PD and improve the treatment plan during the perioperative period. Adequate subacromial debridement may be necessary to reduce the incidence of joint stiffness. Effective advocacy of intensive physiotherapy for patients in the perioperative period was imperative, as well as for preoperative identification of whether the patient was in progress.

In view of the absence of general consensus in the previous studies, lower-level evidence of existing study protocols, larger follow-up investigations are essential to explore the interaction of PD on the perioperative outcomes in ARCR. Several noteworthy limitations must be acknowledged in the current study. First, as a retrospective study, there were many, inevitably, inherent limitations in the course of research. Second, this study only provided mid-term results at 2 years postoperatively, and long-term results remained unclear. Third, the target group was focused only on patients with mild to moderate PD (H&Y stage I-II) and small- to large-sized tears. We lacked sufficient cases to investigate the association between clinical outcomes after ARCR and disease progression in PD statistically. Our study did not obtain sufficient postoperative imaging to determine if poorer outcomes in patients with PD were associated with differences in the anatomical integrity of the tendon. Therefore, the above criteria may limit the generalization and comprehensiveness of the findings and conclusions.

## Conclusion

Patients with PD experience significantly more pain, resulting in worse functional shoulder outcomes and report consistently reduced mental and physical health. Physician should be involved in the preoperative counseling to help slow down the deterioration of PD, which will ameliorate and stabilize the prognosis of ARCR. Further high-grade evidence-based investigations are warranted for accurately assess the risks and benefits of ARCR. Therefore, the decision of ARCR in patients with PD should be thoroughly assessed in light of the patients’ psychological expectations and physical conditions.

## Data Availability

The datasets used and/or analyzed during the current study are available from the corresponding author on reasonable request.

## Abbreviations

ARCR: Arthroscopic rotator cuff repair
PD: Parkinson’s Disease
ROM: Range of motion
WORC: Western Ontario Rotator Cuff Index
CMS: Constant-Murley Score
UCLA: University of California, Los Angeles
PSQI: Pittsburgh Sleep Quality Index
HADS: Hospital Anxiety and Depression Scale
VAS: Visual analog scale
PSM: Propensity score matching
SNpc: Substantia nigra pars compacta
MRI: Magnetic resonance imaging
H&Y: Hoehn and Yahr
